# Cloning of somatolactin alpha, beta forms and the somatolactin receptor in Atlantic salmon: Seasonal expression profile in pituitary and ovary of maturing female broodstock

**DOI:** 10.1186/1477-7827-6-42

**Published:** 2008-09-15

**Authors:** Susana Benedet, Björn Thrandur Björnsson, Geir Lasse Taranger, Eva Andersson

**Affiliations:** 1 Department of Zoology/Zoophysiology, University of Gothenburg, Box 463, 40530, Gothenburg, Sweden; 2 Institute of Marine Research, PO Box 1870 Nordnes, 5817 Bergen, Norway

## Abstract

**Background:**

Somatolactin (Sl) is a fish specific adenohypophyseal peptide hormone related to growth hormone (Gh). Some species, including salmonids, possess two forms: Sl alpha and Sl beta. The somatolactin receptor (slr) is closely related to the growth hormone receptor (ghr). Sl has been ascribed many physiological functions, including a role in sexual maturation. In order to clarify the role of Sl in the sexual maturation of female Atlantic salmon (Salmo salar), the full length cDNAs of slr, Sl alpha and Sl beta were cloned and their expression was studied throughout a seasonal reproductive cycle using real-time quantitative PCR (RTqPCR).

**Methods:**

Atlantic salmon Sl alpha, Sl beta and slr cDNAs were cloned using a PCR approach. Gene expression of Sl alpha, SL beta and slr was studied using RTqPCR over a 17 month period encompassing pre-vitellogenesis, vitellogenesis, ovulation and post ovulation in salmon females. Histological examination of ovarian samples allowed for the classification according to the degree of follicle maturation into oil drop, primary, secondary or tertiary yolk stage.

**Results:**

The mature peptide sequences of Sl alpha, Sl beta and slr are highly similar to previously cloned salmonid forms and contained the typical motifs. Phylogenetic analysis of Atlantic salmon Sl alpha and Sl beta shows that these peptides group into the two Sl clades present in some fish species. The Atlantic salmon slr grouped with salmonid slr amongst so-called type I ghr. An increase in pituitary Sl alpha and Sl beta transcripts before and during spawning, with a decrease post-ovulation, and a constant expression level of ovarian slr were observed. There was also a transient increase in Sl alpha and Sl beta in May prior to transfer from seawater to fresh water and ensuing fasting.

**Conclusion:**

The up-regulation of Sl alpha and Sl beta during vitellogenesis and spawning, with a subsequent decrease post-ovulation, supports a role for Sl during gonadal growth and spawning. Sl could also be involved in calcium/phosphate mobilization associated with vitellogenesis or have a role in energy homeostasis associated with lipolysis during fasting. The up-regulation of both Sl alpha and Sl beta prior to fasting and freshwater transfer, suggests a role for Sl linked to reproduction that may be independent of the maturation induced fasting.

## Background

Somatolactin (Sl) is a peptide hormone exclusive to fish, and belongs to the class I helical cytokine family which includes growth hormone (Gh), prolactin (Prl) and leptin [1-3]. Two distinct forms coded by separate genes and produced in different cells in the *pars intermedia *of the pituitary have been isolated: Sl alpha (Slα) which is present in all fish, and Sl beta (Slβ) which has only been found in a limited number or species spending all or part of their lifecycle in freshwater [4]. The Sl receptor (slr), recently characterized in masu salmon (*Oncorhynchus masou*) [5], is closely related to the Gh receptor (ghr) with which it shares many structural features. The degree of sequence similarity has led to categorization of all known putative fish ghrs and slrs into two types of ghr; type I and type II [6]. The masu salmon slr appears more closely related to the type I ghr than the type II ghr.

Like Gh and Prl, Sl is thought to have multiple functions including a undefined role in reproduction. In salmonids, an increase in plasma Sl levels concomitant with final gonadal growth has been found [7,8]. Also, in mature rainbow trout (*O. mykiss*) and chinook salmon (*O. tshawytscha*), plasma Sl levels are higher than in immature fish [9,10], and in chum salmon (*O. keta*), Sl mRNA expression is elevated during sexual maturation [11]. Further, immunocytochemistry indicates an activation of somatolactotrophs in sexually maturing and spawning sockeye (*O. nerka*), chum and chinook salmon [12,13]. However, other studies have found no correlation between plasma Sl and final gonadal maturation [14], and therefore, the specific actions of Sl during salmonid reproduction are not clear.

The hormone has been suggested to act during early oogenesis [15], gonadal maturation [7,8] and gonadal steroid biosynthesis [16]. Sl has also been proposed to act as a facilitator of oocyte maturation through its regulation of lipid metabolism which in salmonids is believed to be linked to suppression of organ accumulation of fat. This is based on the high amount of intraperitoneal fat and an enlarged liver with high glycogen and fat content found in the cobalt rainbow trout mutant which lacks the pituitary *pars intermedia *[17-19], the high triglyceride and cholesterol content in liver and muscle of Sl deficient mutant medaka *color interfere (ci*) (*Oryzias latipes*) [20], and the high expression of *slr *transcripts in liver and visceral fat in coho salmon and medaka [5,20].

The current study has two major aims: 1) to obtain the full cDNA sequence of Slα, Slβ and slr in Atlantic salmon (*Salmo salar*) and to phylogenetically analyse their structure in relationship to other fish Sl, slr and ghr sequences, and 2) to begin to clarify the role of these genes in late oocyte female maturation. To do this, gene expression was measured in the ovary (*slr*) and pituitary (*Slα, Slβ*) using RTqPCR in 3+ year old female salmon during a 17-month period comprising late oocyte growth, ovulation and post ovulation. Atlantic salmon was chosen as an important model salmonid species of commercial value.

## Methods

### Sequencing of Atlantic salmon Slα and Slβ

Total RNA was extracted from six pooled pituitaries of juvenile Atlantic salmon stored in RNAlater (Ambion, Austin, TX) using the RNeasy Mini kit (Qiagen, Germantown, MD) with treatment with RNase free DNase I (Qiagen). Three micrograms of this total RNA was reverse transcribed into cDNA using Power Script Reverse Transcriptase (Clontech, Palo Alto, CA) using oligo (dT)_12_. Two primers based on the rainbow trout Slα cDNA [21] were used to obtain Atlantic salmon Slα open reading frame (ORF) of 721 nucleotides: SlαF1 and SlαR1 (Table 1). A primer pair based on the rainbow trout Sl-like protein (rtSLP), the putative Slβ form [22], was used to obtain the partial Atlantic salmon Slβ ORF of 627 nucleotides: SlβF1 and SlβR1 (Table 1). PCR was carried out for 30 cycles of 94°C for 15 s, 55°C for 30 s and 68°C for 60 s using Platinum Pfx DNA polymerase (Invitrogen, Carlsbad, CA). The resulting bands, 720 bp for Slα and 610 bp for Slβ as seen on 1.2% agarose gel, were purified and directly sequenced by an ABI capillary sequencer (MWG Biotech, Ebersberg, Germany). The corresponding 5' and 3' ends were obtained by RACE with the SMART RACE cDNA Amplification Kit (Clontech) using gene specific primers: Slα5RACErev, Slα3RACEfor, Slβ5RACErev and Slβ3RACEfor (Table 1). All RACE products were gel purified, subcloned and sequenced and yielded the complete Atlantic salmon Slα and Slβ cDNA.

**Table 1:** Primer sequences for PCR cloning.

**Primers for sequencing Atlantic salmon slr:**
slrF1: 5'-CGTGCCAGAGATTCCCAATAAAGAGTCAAC-3'
slrR1: 5'-GCTTGTCTGTTCTCCTCTCCTCCTC-3'
slrF3: 5'-GACTTCTATGCACAGGTCAGCGATGTGACGC-3'
slrR2: 5'-GAAGATGATGAGCATGAGGAGAATGGCCACGC-3'
slrF7: 5'-CACAACAACCACCACACAAACCATGG-3'
slrR9: 5'-CAAGTAGAACAAGGCTGTCTGTGATG-3'
slr5RACErev: 5'-TCAGAGTGGAGCCGCACACAGTATTTGATCC-3'
slr3RACEfor: 5'-TACCTGTTCCCCACAACCCCCCTCCAAG-3'

**Primers for sequencing of Atlantic salmon Slα:**

SlαF1: 5'-CACCATGAACATGATGCAAGTCATGCAG-3'
SlαR1: 5'-GGGTGGGYATAGTTGTTCTCTAATGAAG-3'
Slα5RACErev: 5'-CACAAAGTATGATGTTGCCCTGCTCGTCCTTAC-3'
Slα3RACEfor: 5'-TGGACGATGATATGCTGACCACCTCCTACTAC-3'

**Primers for sequencing of Atlantic salmon Slβ:**

SlβF1: 5'-CACCATGGAGTGTCAGGATCC-3'
SlβR1: 5'-ACTGGAGGGACTCTGCTATAAAAG-3'
Slβ5RACErev: 5'-GTGCACGCGTTCCCTGTTTGAGTCAACG-3'
Slβ3RACEfor: 5'-GAGCCTGGCAGACTACCCTGTGCATTTTG-3'

### Sequencing of Atlantic salmon slr

Total RNA was extracted from about 30 mg of liver of adult Atlantic salmon and this and production of cDNA was carried out as described above for the two Sl forms. Three overlapping pairs of primers were designed based on the masu salmon slr [5] and differing from Atlantic salmon ghr and trout prl receptor; pair one slrF1 and slrR1, pair two slrF3 and slrR2 and pair three slrF7 and slrR9 (Table 1). cDNA was amplified by PCR with Advantage 2 Polymerase (Clontech). Cycling consisted in 35 cycles of 95°C for 30 s, 61°C for 45 s and 72°C for 90 s. The resulting products yielded bands of the expected sizes. The bands were purified from the gel, subcloned into pGEM-T Easy Vector (Promega, Madison, WI) and sequenced, resulting in 2064 nucleotides of sequence. The full Atlantic salmon slr was obtained using the SMART RACE cDNA Amplification Kit using gene specific primers: slr5RACErev and slr3RACEfor (Table 1).

### Phylogenetic analysis

Protein sequence alignments were carried out with ClustalW (1.83) software [23]. Phylogenetic analysis of putative fish slr and ghr sequences and Sl sequences were made by the maximum likelihood method using the PHYLIP 3.6a3 package [24]. Branch lengths show genetic change and bootstrap values are shown as % on the branches (100 datasets). The analysis using parsimony and distance-matrix methods such as UPGMA and neighbour joining generated trees of similar topology and lower bootstrap values.

### Measurement of ovarian *slr *and pituitary *Slα *and *Slβ *gene expression

Atlantic salmon were raised from fertilized eggs at the Matre Aquaculture Research Station, Matredal, Norway. After smoltification in their second spring (1+ aged fish), the mixed population was transferred to seawater netpens and fed *ad lib *with a commercial dry pelleted feed. Three year old females were sampled on 15 occasions during a 17-month period from August 2004 to December 2005, a month after spawning. The fish were held in seawater netpens until May 2005, when they were moved to freshwater tanks just after the May sampling. In the freshwater tanks, the fish were subject to simulated natural photoperiod and ambient water temperature. They stopped voluntary feeding after transfer to the tanks and fasted until the end of the experiment. Thus, the project covers the period of oocyte maturation from early secondary growth (oil-drop stage) through vitellogenesis, ovulation and post ovulation.

At approximately monthly intervals, six to ten females were sampled under metomidate anesthesia (6 mg/L, Syndel, Victoria, B.C.). All fish were treated and killed according to Norwegian National Legislation for laboratory animals. The animals were measured and weighed. Blood was drawn from the caudal vein, plasma obtained by centrifugation and quick frozen in liquid nitrogen and stored at -80°C until analysis. Then the fish were immediately sacrificed by sectioning the *medulla oblongata*, gonads excised and weighted to calculate gonadosomatic index (GSI; gonad weight as a percentage of total body weight). Ovary samples consisting of a piece of ovarian lamella and whole pituitaries were quick frozen in liquid nitrogen and stored in -80°C until analyzed for RNA extraction. Plasma levels of 17β-estradiol (E_2_) and testosterone (T) were determined by ELISA [25].

To assess the expression profile in the ovaries, total RNA was isolated from about 60 mg frozen tissue using the FastPrepPro RNA Isolation System (Qbiogene, Illkirch, France) according to the manufacturer's instructions. For pituitaries, total RNA from individual pituitaries was obtained with the PARIS™ Kit (Ambion) in combination with the FastPrep RNA Isolation System (Qbiogene) according to the manufacturer's instructions. Quantity and quality of the RNA were determined by UV absorbance at 230, 260 and 280 nm. Total RNA was treated with DNase I (Promega, Southampton, UK) and the integrity of the RNA was verified with a Bioanalyzer 2100 expert system (Agilent Technologies, Palo Alto, CA, USA). Reverse transcription into cDNA took place in the presence of the Moloney murine leukaemia virus reverse transcriptase using a Reverse Transcription Core kit (RT-RTCK-05, Eurogentec, Searing, Belgium) according to the manufacturer's instructions, with 500 ng total RNA in a 30 μl reaction volume. The cDNA was diluted 10-fold with nuclease-free water.

Relative gene expression of *Slα*, *Slβ *and *slr *was measured by TaqMan PCR assays in duplicate, using 96-well optical plates on an ABI PRISM 7700 Sequence Detector, using default settings. For each 25 μl PCR reaction, 5 μl cDNA was mixed with 200 nM fluorogenic probe, 900 nM sense primer, 900 nM antisense primer in 1xTaqMan Universal PCR Master Mix (Applied Biosystems (ABI), Foster City, CA). Atlantic salmon *elongation factor 1 alpha (ef1α) *[GenBank:AF321836] was used as a house-keeping reference gene after validating its stable temporal expression. The *slr *primer pair and probe were based on those used to study *slr *in masu salmon [5] and were located in the intracellular domain (Table 2). The specificity of all primer pairs and corresponding probes was validated by PCR amplification, sequencing of products and melting curve analysis using SYBR^® ^Green (Invitrogen). The efficiency of the systems was validated by running dilution series which gave ΔCt/log cDNA curves with slopes <0.1 Relative gene expression was calculated using the comparative method (ΔΔCt) as described in detail previously [26] where the target gene is normalized to the internal reference gene (*ef1α*) and target amount given relative to the calibrator (initial August sample).

**Table 2:** Primers and probes for RT qPCR.

ef1α forward: 5'-CCCCTCCAGGACGTTTACAAA-3'
ef1α reverse: 5'-CACACGGCCCACAGGTACA-3'
ef1α probe: 5'-ATCGGTGGTATTGGAAC-3'
slr forward: 5'-CAGCACTGCTTAAGAAGGGAAAG-3'
slr reverse: 5'-TGGAGAGCCCCCATACCA-3'
slr probe: 5'-CCACTCAGGATGAAGTTCAGCTCGTCCA-3'
Slα forward: 5'-GAAATCCAGCAGATCTCAGACAAG-3'
Slα reverse: 5'-GTACACCAATGGCTCAATCCAG-3'
Slα probe: 5'-TCCTCCACTCTGTCCTAATTCTGGTCCAGT-3'
Slβ forward: 5'-CATCCCCACTTCCAGAAGTG-3'
Slβ reverse: 5'-AGGAACAGAATGGAGTGCAAAAG-3'
Slβ probe: 5'-TCCAAGAAAACTCTGACAAGTG-3'

### Histological analysis of oocyte maturation

At the time of sampling, a piece of ovarian lamella was isolated by transversal cuts with a scalpel blade from each female and fixed in Bouin's fixative for histological analysis. Fixed samples were transferred to ethanol (70%), and subsequently dehydrated and embedded in paraffin according to conventional techniques. Histological examination was carried out as described previously [27] using 5 μm sections after hematoxylin-eosin staining. Animals considered as immature, i.e. showing primary growth phase follicles only, were not found in the present study. As salmon females are not fully synchronous in their sexual maturation, females sampled at a certain date may have reached different levels of maturity. Thus, it is important not only to analyze the data in relation to date, but also in relation to maturational status. This was determined for each sampled female according to the most advanced type of follicles as follows: (i) oocytes with cortical alveoli and perinuclear oil droplets (oil drop), or (ii) showing follicles with oocytes in true vitellogenesis. The latter were further differentiated in oocytes showing a limited number of small eosinophilic yolk globules in the periphery of the oocyte (primary yolk stage), with numerous but still small yolk globules distributed throughout the oocyte (secondary yolk stage), or with oocytes filled with large yolk globules (tertiary yolk stage). This analysis was carried out until all ovaries were found to have reached tertiary yolk state (June).

Both in the time-based (n = 6–10) and in the maturation-based (n = 14–24) analyses, the data were tested for homogeneity of variance using Levene's test, and where appropriate, the data were log-transformed or arctan-transformed before analysis of variance (ANOVA with P < 0.0166 to compensate for comparison of three variables) using SPSS version 15.0 (SPSS Inc., Chicago, IL). Tukey's multiple means comparison test was performed as post-hoc test. Correlation analysis using two-tailed Spearman's ρ was carried out at each sampling date and for each maturational category. All data are presented as means ± standard error of the mean (SEM).

### *Slα*, *Slβ *and *slr *gene expression tissue distribution

In order to determine expression levels of *Slα*, *Slβ *and *slr *mRNA, a series of tissue samples from 3 immature post-smolt females from the same strain that was used in the maturation expression study where collected, flash frozen and stored at -80°C. Total RNA was extracted as described above and cDNA obtained from pituitary, brain, gill, liver, ovary, head kidney, kidney, spleen, muscle, intestine, pylorous and heart. Gene expression was determined by RTqPCR using the same primer pairs and cycling conditions as described above.

## Results

### Atlantic salmon Slα and Slβ

**Figure 1:**
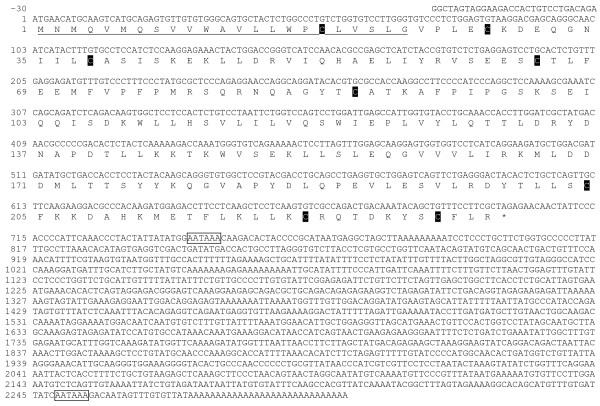
**Atlantic salmon Slα cDNA sequence [GenBank:**EU255779]. Nucleotides and amino acids are positively numerated starting with the initiation methionine. Signal peptide (underlined with solid line); Cysteine residues c; Stop (*); Polyadenylation signal (boxed).

The cloning of Atlantic salmon Slα yielded the complete cDNA of 2331 base pairs (bp) that included 30 bp of 5'untranslated region (UTR), 699 bp of open reading frame (ORF), and a long 3'UTR of 1602 bp (Fig. 1). The ORF translated into 232 amino acids (aa) including a 23 aa signal peptide and 209 aa mature protein with a predicted molecular weight (MW) of 24.2 KDa. There are eight cysteine residues in the full-length protein of which seven are present in the mature protein. There are two polyadenylation signals.

**Figure 2:**
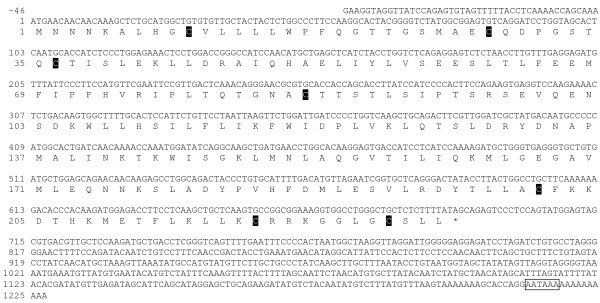
**Atlantic salmon Slβ cDNA sequence [GenBank:**EU255780]. Nucleotides and amino acids are positively numerated starting with the initiation methionine. Signal peptide (underlined with solid line); Cysteine residues c; Stop (*); Polyadenylation signal (boxed).

Cloning of Atlantic salmon Slβ yielded the complete cDNA of 1273 bp that included 46 bp of 5'UTR, 690 bp of ORF, and a 3'UTR of 537 bp (Fig. 2). The ORF translates into 229 aa including a 23 aa signal peptide and 206 aa mature protein with a predicted MW of 23.4 KDa. Seven cysteine residues are present in the full-length protein of which six are present in the mature protein. One potential polyadenylation signal was found.

**Figure 3:**
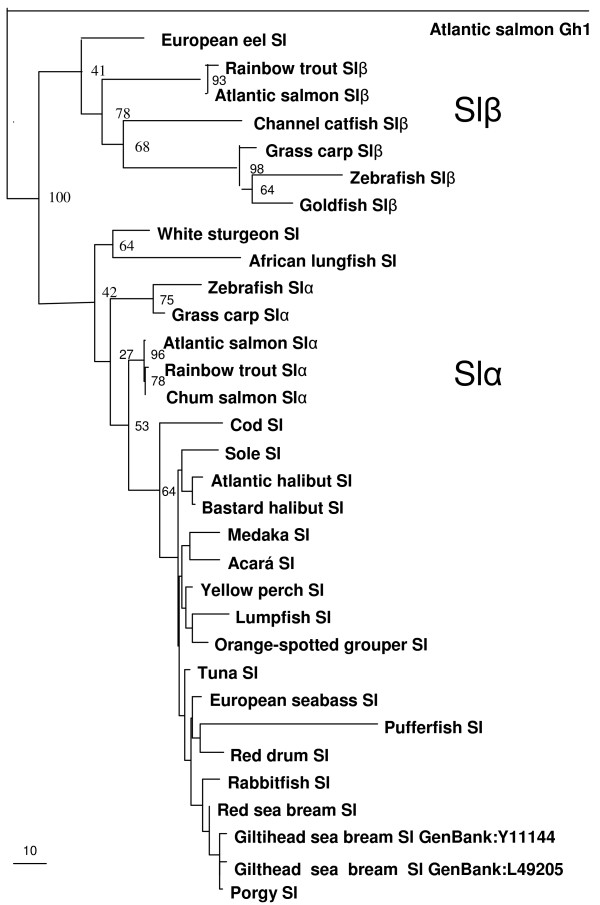
**Phylogenetic analysis of Sl sequences**. Phylogram of fish Sl amino acid sequences showing genetic change made by maximum likelihood method. Atlantic salmon Gh1 (*Salmo salar *[GenBank:X14305]) is the rooting out-group and bootstrap values as % are shown on the branches (100 datasets). European eel (*Anguilla Anguilla *[GenBank:U633884]), rainbow trout Slβ (*Oncorhynchus mykiss; *[22]), Atlantic salmon Slβ [GenBank:EU255780], channel catfish Slβ (*Ictalurus punctatus *[GenBank:AF267991]), grass carp Slβ (*Ctenopharyngodon idella *[GenBank:EF372075]), zebrafish Slβ (*Danio rerio *[GenBank:AY221126]), goldfish (*Carassius auratus *[GenBank:U72940]), white sturgeon (*Acipenser transmontanus *[GenBank:AB017200]), African lungfish (*Protopterus annectens *[GenBank:AB017766]), zebrafish Slα [GenBank:AY376857], grass carp Slα [GenBank:EF372074], rainbow trout Slα ([21]), chum salmon Slα (*Oncorhynchus keta *[GenBank:D10638]), cod (*Gadus morhua *[GenBank:D10639]), Senegalese sole (*Solea senegalensis *[GenBank:U06753]), Atlantic halibut (*Hippoglossus hippoglossus *[GenBank:L02117], Bastard halibut (*Paralichthys olivaceus *[GenBank:M33696]), medaka (*Oryzias latipes *[GenBank:NM_001104790]), acarα (*Cichlasoma dimerus *[GenBank:EF192603]), yellow perch (*Perca flavescens *[GenBank:AY332490]), lumpfish (*Cyclopterus lumpus *[GenBank:L02118]), orange spotted grouper (*Epinephellus coioides *[GenBank:AY169406]), tuna (*Thunnus thynnus *[GenBank:AB222036]), European sea bass (*Dicentrarchus labrax *[GenBank:AJ277390]), pufferfish (*Tetraodon miurus *[GenBank:AF253066]), red drum (*Sciaenops ocellatus *[GenBank:AF062520]), rabbitfish (*Siganus guttatus *[GenBank:AB026186]), red sea bream (*Pagus major *[GenBank:AB219244), black porgy (*Acanthopagrus schlegelii *[GenBank:AY714370]).

The phylogenetic tree of the Sls (Fig. 3) shows the two well-differentiated clades of Slα and Slβ. Up to date, seven species have beeen found to have putative Slβ and all these species spend all or part of their lives in freshwater, none is exclusively marine. The degree of similarity between Atlantic salmon Slα and Slβ was 54% for the protein and 65% for the coding DNA. The similarity of Slα and Slβ with Atlantic salmon Gh1 was 26% and 23%, respectively, and with Atlantic salmon Prl, 19% and 17%, respectively. Both Atlantic salmon Slα and Slβ were 96% similar to the rainbow trout and chum salmon Sls. The degree of similarity between the Slβ of different fish species is less (42–53%) than for Slα (54–81%).

### Atlantic salmon Sl receptor (slr)

**Figure 4:**
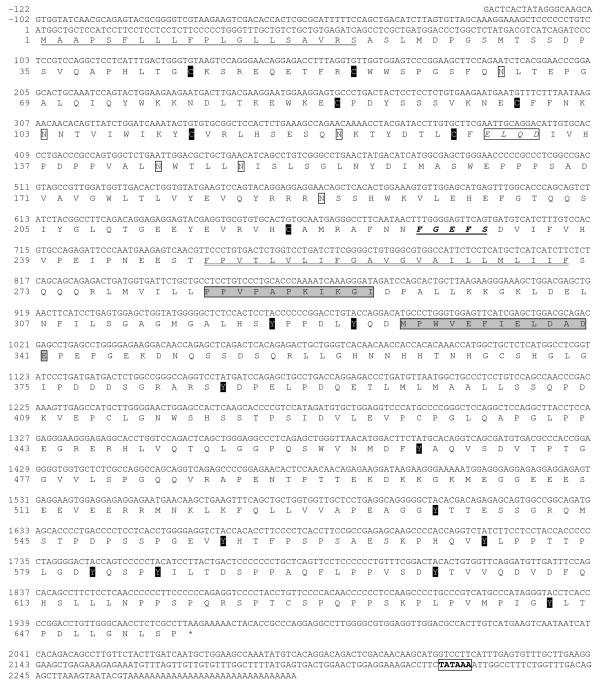
**Atlantic salmon slr cDNA sequence [GenBank EU242505]**. Nucleotides and amino acids are positively numerated starting with the initiation methionine. Signal peptide (underlined); Transmembrane domain (underlined with double line); Site B motif (box); FGEFS motif (bold italics underlined); Extracellular cysteine residues (C); Potential extracellular N-glycosilation sites (open boxes); Potential intracellular tyrosine phosphorylation sites (Y); Box I and Box II (shaded boxes); Stop (*); Polyadenylation signal (boxed).

The Atlantic salmon slr cDNA sequenced consists of 2,369 bp, including a 5'UTR of 77 bp and 3'UTR of 324 bp (Fig. 4). The ORF of 1968 nucleotides translates into 655 aa, including a 20 aa signal peptide, 228 aa extracellular domain (ECD), 23 aa transmembrane domain, and 384 aa intracellular domain (ICD). The predicted MW of the mature receptor is 70.5 KDa. The cloned Atlantic salmon slr shares features and domains present in the ghr: the typical FGEFS motif, and slightly different Box 1 (PPVPAPKIKGI) and Box 2 (DLYQDMPWVEFIELD) in the ICD. In addition to the five cysteine residues present in the ECD of most teleost ghr and prlr, it has two additional cysteine residues not present in most type II ghr, making a total of seven ECD cysteines. It has four, instead of five, potential N-linked glycosylation sites in the ECD and 11 intracellular tyrosine residues whereas Atlantic salmon ghr1 and ghr2 have only seven. The Atlantic salmon slr is 61 and 65 aa longer than the Atlantic salmon ghr1 and ghr2, respectively, and the differences are mainly found in the ICD signaling region.

**Figure 5:**
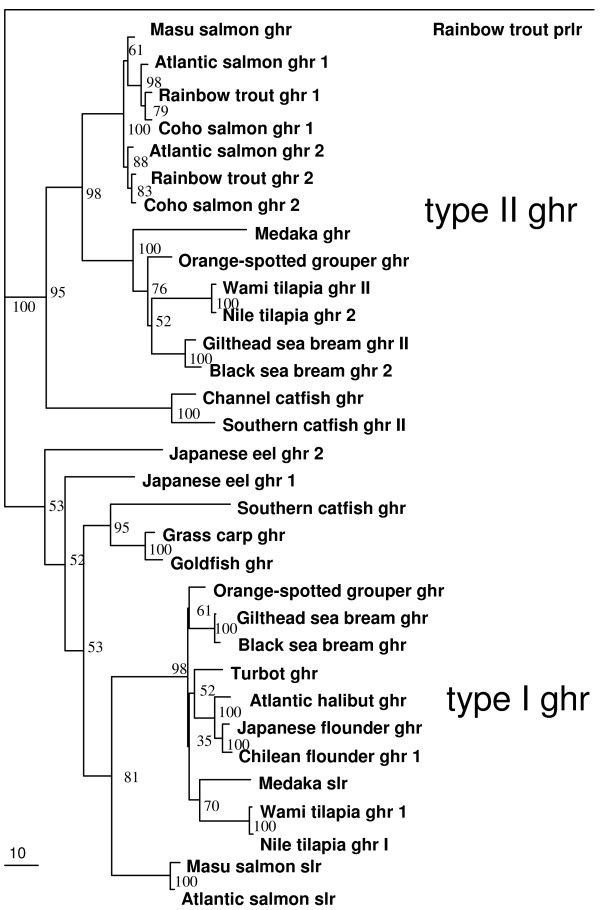
**Phylogenetic analysis of putative slr and ghr sequences**. Phylogram of fish slr and ghr amino acid sequences showing genetic change made by maximum likelihood method. Rainbow trout prlr (*Oncorhynchus mykiss *[GenBank:AF229197]) is the rooting out-group and bootstrap values as % are shown on the branches (100 datasets). Masu salmon ghr (*Oncorhynchus masou *[GenBank:AB071216]), Atlantic salmon ghr1 (*Salmo salar *[GenBank:AY462105]), Rainbow trout ghr1 (*Oncorhynchus mykiss *[GenBank:AY861675]), coho salmon ghr1 (*Oncorhynchus kisutch *[GenBank:AF403539]), Atlantic salmon ghr2 [GenBank:DQ163908], Rainbow trout Ghr2 [GenBank:AY751531], coho salmon ghr2 [GenBank:F403540], medaka ghr (*Oryzias latipes *[GenBank:DQ010539]), orange spotted grouper ghr (*Epinephelus coioides *[GenBank:EF052273]), Wami tilapia ghrII (*Oreochromis urolepis hornorum *[GenBank:EF371467]), Nile tilapia ghrII (*Oreochromis niloticus *[GenBank:EF052862]), Gilthead sea bream ghrII (*Sparus aurata *[GenBank:AY573601]), black sea bream ghrII (*Acanthopagrus schlegelii *[GenBank:AY662334]), channel catfish ghr (*Ictalurus punctatus *[GenBank:DQ103502]), Southern catfish ghrII (*Silurus meridionalis *[GenBank:AY973231]), Japanese eel ghr2 (*Anguilla japonica *[GenBank:AB180477]), Japanese eel ghr1 [GenBank:AB180476], Southern catfish ghr [GenBank:AY336104], grass carp ghr (*Ctenopharyngodon idella *[GenBank:AY283778]), goldfish ghr (*Carassius auratus *[GenBank:AF293417]), orange-spotted grouper ghr [GenBank:EF052273], gilthead sea bream ghr [GenBank:AF438176], black sea bream ghrI [GenBank:AF502071], turbot ghr (*Scophthalmus maximus *[GenBank:AF352396]), Atlantic halibut ghr (*Hippoglossus hippoglossus *[GenBank:DQ062814]), Japanese flounder ghr (*Paralichthys olivaceus *[GenBank:AB058418]), Chilean flounder ghrI (*Paralichthys adspersus *[GenBank:EU004149]), medaka slr [GenBank:DQ002886], wami tilapia ghrI [GenBank:EF371466], Nile tilapia ghrI [GenBank:AY973232], masu salmon slr [GenBank:AB121047], Atlantic salmon slr [GenBank:EU242505].

The aa sequence of the Atlantic salmon slr is most similar (94%) to the masu salmon slr, while sharing 44%- 43% aa and 59% nucleotide similarity with Atlantic salmon ghr isoforms 1 and 2 (86% alike), and 17% similarity with rainbow trout prlr. A phylogenetic analysis (Fig. 5) shows that Atlantic salmon slr protein shares greater similarity with the so-called type I ghr (43–56% aa similarity with non-salmonids) than with type II ghr (35–43% aa similarity with non-salmonids). The similarity of Atlantic salmon slr with the putative medaka slr is 49%.

### Measurement of ovarian *slr *and pituitary *Slα *and *Slβ *gene expression

At the beginning of the 17 month study period, in August 2003, the fish had a mean body weight of 4.3 kg ± 0.3 (mean ± SEM), a mean length of 69.7 cm ± 1.5, and a mean GSI of 0.22% ± 0.02. At the last sampling in December 2004, the mean body weight was 9.4 kg ± 0.4 and the mean length was 94.0 cm ± 1.5. Somatic weight (body weight – gonad weight) increased steadily until May (11.5 kg ± 0.5) and afterwards experienced a decline which was slight at first (May to August: -8.58%) but sharp afterwards (August-to spawning in Oct-Nov: -26.6%). Mean somatic weight in October, just before spawning, was 7.89 kg ± 0.4. GSI was under 1% up until April, doubling in May to 1.86% and rising steadily until August when it took off almost tripling the July value to 7.89%. Mean GSI in October, just before spawning, was 20.0% ± 0.6.

**Figure 6:**
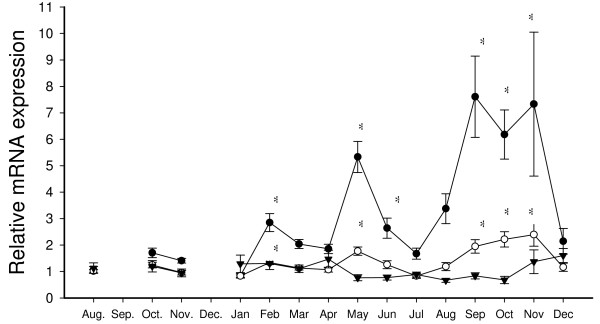
**Relative mRNA expression of *Slα *(○) and *Slβ *(●) in the pituitary and *slr *(▼) in the ovary of Atlantic salmon female broodstock from August 2003 to December 2004**. Each point represents mean ± SEM of n = 6–10 per date and * denotes significance at P < 0.0166. Relative transcription levels were normalized to *ef1α *and compared to the initial August sample.

Ovarian *slr *relative gene expression was not significantly up- or down-regulated during the 17-month study (Fig. 6). Pituitary expression of *Slα *had a 29-fold higher expression than *Slβ*, but *Slβ *showed stronger up- and down-regulation. Both *Slα *and *Slβ *increased significantly before and during spawning (September, October and November); two fold for *Slα *and sevenfold for *Slβ*. There was a subsequent down-regulation to baseline levels during ovarian post ovulation in December 2005. A significant transient two-fold (*Slα*) and five-fold (*Slβ*) increase in expression was observed in May prior to transfer from seawater to fresh water. Another significant, but transient, increase in *Slα *and *Slβ *occurred in February. *Slα *and *Slβ *expression were found to correlate significantly with each other throughout the period of March to October (n = 6–10; ρ = 0.68–0.85; 4 months P < 0.01 and 4 months P < 0.05). *Slα *and *Slβ *were found to correlate negatively with E_2 _(P < 0.01) and T (P < 0.05) during June, when both were downregulated. *Slβ *correlated positively with E_2 _in August and November whereas *Slα *correlated positively with E_2 _in December (P < 0.05). In the period from July to December (n = 46), *Slα *and *Slβ *correlated with E2 (P < 0.05), T (P < 0.01), gonad weight (P < 0.01) and GSI (P < 0.01).

### Ovarian *slr *and pituitary *Slα *and *Slβ *gene expression related to histological classification of oocyte maturation

**Figure 7:**
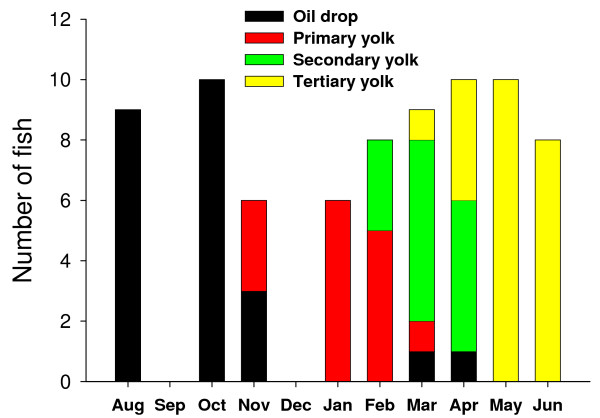
**Distribution of follicle stage categories in time**. Oil drop n = 23; Primary yolk n = 14; Secondary yolk n = 15; Tertiary yolk n = 24.

**Figure 8:**
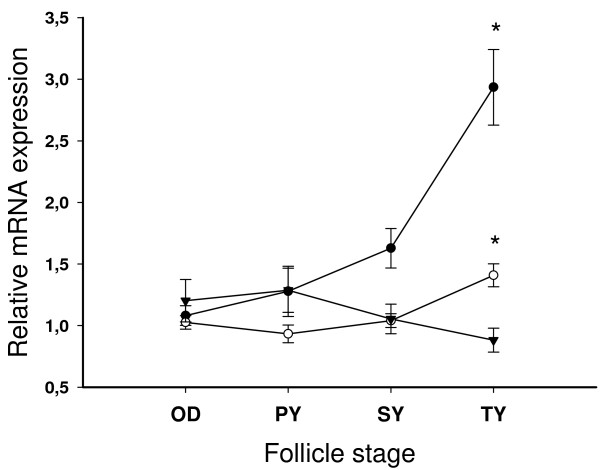
**Relative mRNA expression of *Slα *(○) and *Slβ *(●) in the pituitary and *slr *(▼) in the ovary o f Atlantic salmon female broodstock according to follicle stage**. OD = oil drop, PY = primary yolk, SY = secondary yolk, TY = tertiary yolk. Each point represents mean ± SEM of n = 14–24 per stage and * denotes significance at P < 0.0166. Relative transcription levels were normalized to *ef1α *and compared to the initial OD stage.

The analysis and classification of the ovarian histological sections from August 2003 to June 2004 showed a significant asynchrony of the maturational process with some females reaching maturation milestones 3–4 months earlier than others (Fig. 7). In May 2004, all the fish had reached the tertiary yolk state. As for the time-based analysis, ovarian *slr *relative gene expression was not significantly up- or down-regulated during the period of oocyte maturation from oil drop to tertiary yolk stage (Fig. 8). Pituitary expression of both *Slα *and *Slβ *were upregulated during the tertiary yolk stage, especially *Slβ*. *Slα *and *Slβ *correlated with each other at all oocyte stages (n = 14–24; ρ = 0.67–0.88; P < 0.01).

### *Slα*, *Slβ *and *slr *gene expression tissue distribution

**Figure 9:**
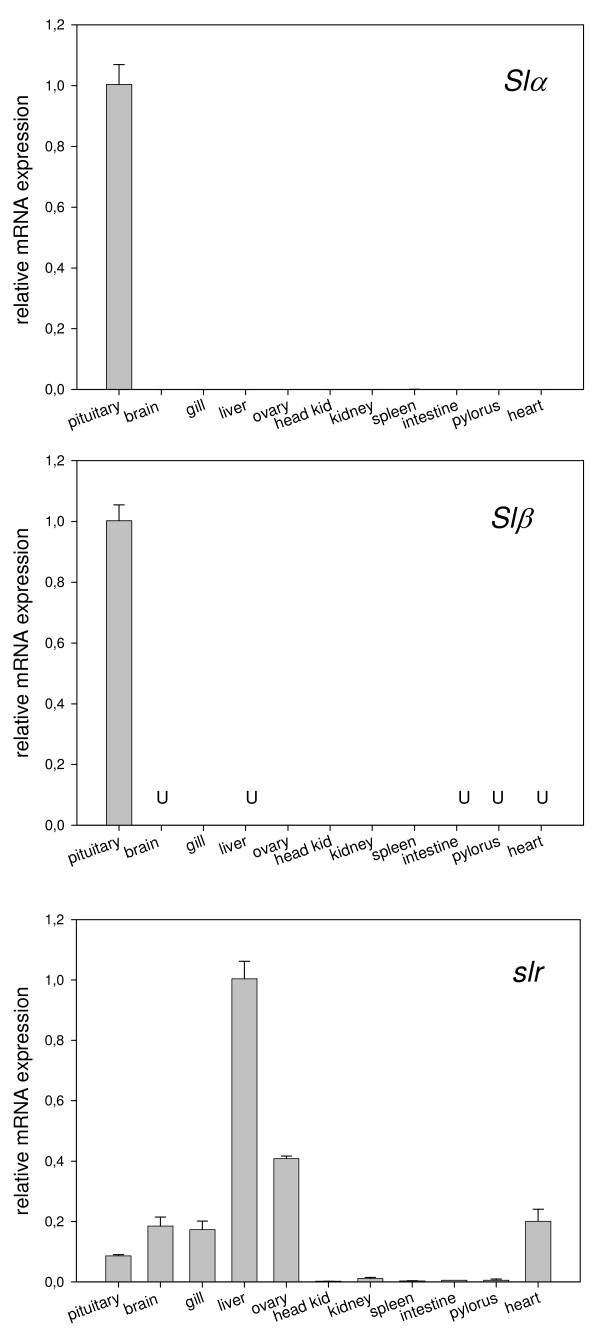
**Tissue distribution of *Slα*, *Slβ *and *slr *in immature female Atlantic salmon post-smolts**. Each column represents mean ± SEM of n = 1–3 per tissue and gene. Relative transcription levels were normalized to *ef1α *and compared to pituitary levels in the case of *Slα *and *Slβ *and liver levels in the case of *slr*. U means transcripts were undetected.

Fig. 9 shows the expression levels of *Slα*, *Slβ *and *slr *mRNA as determined by RTqPCR in a series eleven tissues of immature female Atlantic salmon post-smolts. In some of the samples the expression levels of *Slα*, and specially *Slβ *(brain, heart, pylorous, intestine, liver) were not detectable so that sample size was n<3. Both *Slα *and *Slβ *transcripts were highest in the pituitary. *Slr *expression level was highest in the liver, followed by ovary, heart, brain, gill and pituitary.

## Discussion

### Structure and characteristics of Atlantic salmon Slα and Slβ

The Atlantic salmon Slα and Slβ bear high degree of aa similarity to previously cloned chum salmon and rainbow trout Sl forms [3,21,22]. When analyzed phylogenetically, each Atlantic salmon Sl form groups in a distinct Slα or Slβ clade, as previously established for other species [4]. Slα and Slβ are paralogous and are encoded by two separate genes [4]. Although three polyadenylation sites can be found in salmonid Slα, there appears to be only one 2.4 kb mRNA transcript [3]. Slα has been found in all fish species studied and its structure is more conserved among fish groups than the structure of Slβ. This suggests a more central role of Slα in the endocrine regulation of physiological processes in teleost fish. The present data indicate that although Slβ has a 29-fold lower expression level than Slα, the two genes have a very similar expression pattern during sexual maturation. The two Sl forms are produced by different cells in the pituitary gland. Slα is produced in somatolactotrophs in the posterior *pars intermedia *of the pituitary [1,2,18,28,29] that are periodic acid-Schiff (PAS)-negative in salmonids [2], perhaps due to lack of N-glycosylation sites [3]. In gilthead sea bream (*Sparus auratus*), these somatolactotrophs have been found to co-localize with parathyroid hormone related protein (PTHrP) cells and in some instances, both hormones are found in the same cell [30,31]. PTHrP is considered to be a hypercalcemic factor in fish [32]. Slβ is produced by somatolactotrophs in the anterior *pars intermedia *[4]. Atlantic salmon Slβ has the Asn-Lys-Thr site which has been found to be glycosylated in Atlantic cod (*Gadus morhua*) [2] and is also conserved in rainbow trout Slβ (rtSLP) [22], but not in salmonid Slα forms.

Sl has been found in lungfish (*Protopterus annectens*), sturgeon (*Acipenser transmontanus*) and teleost fish. It is therefore assumed to have existed in the ancestral tetrapod, but been lost in the tetrapod lineage [33]. The two Sl forms are thought to have originated during the teleost genome duplication event (3R), after the divergence of the sturgeons. A genome search in *Fugu rubripes*, *Tetraodon nigroviridis *and medaka reveals only the Slα form, so that in some groups, the Slβ form appears to have been lost [4,33]. For the species studied, the intra-species aa similarity between the two forms is relatively low, only 41.8–49.8%. The β form lacks the third cysteine of the seven conserved found in the α form [34] which could reduce its dimerization potential.

Like its sister hormones Gh and Prl, Slα is thought to have multiple functions. The hypothalamic regulation of Sl release appears different from that of Gh or Prl [35]. Some of the functions have been confirmed such as its role in body coloration through xantophore regulation [36-39] and control of lipolysis and energy mobilization [9,18,20,29,40]. Sl also appears to have a role in acid-base balance [41], homeostasis of phosphate [42], sodium [36] and calcium [14,31,43], smoltification [8], stress [9], immune function [44] and in gonadal maturation [15].

Slβ has only been found in a small number of species all of which are either freshwater-living (channel catfish, *Ictalurus punctatus*), the grass carp (*Ctenopharyngodon idella*), zebrafish (*Danio rerio*) and goldfish (*Carassius auratus*) [4,22,45], or spend a part of their life in fresh water within a catadromous (European eel, *Anguilla anguilla*) or an anadromous lifecycle (rainbow trout and Atlantic salmon). The specific roles of Slβ are not clear though Slβ has been found to induce melanosome aggregation in zebrafish [34].

### Structure and characteristics of Atlantic salmon slr

The slr, as the ghr, belongs to the class I cytokine receptor super-family which also includes the prlr and the leptin receptor. These are known to act through JAKs (Janus kinases) and STATs (signal transducers and activators of transcription). The full-length Atlantic salmon slr cloned is very similar (93% aa) to the characterized Masu salmon slr [5]. It possesses basically the same structure and domains as Atlantic salmon ghr isoforms 1 and 2, but shares only 43 and 44% peptide similarity. Some important differences between slr and ghr are observed such as the number and position of extracellular cysteine residues which can form three disulfide bonds in the Atlantic slr and two in ghrs. Slr [5] possess a different site B than teleost ghr, which is believed to be a Gh-binding site in ghr [46]. The ICD is somewhat longer in the slr and has a larger number of tyrosine residues (eleven instead of seven). These are believed to be involved in intracellular signaling along with the Box I and Box II domains, which are also slightly different. The implications of the structural differences between the salmonid slr and ghr could be large in terms of differential specific binding and intracellular signaling.

A phylogenetic analysis of putative fish slr and ghr (Fig. 5) reveals that salmonid slr form a clade amongst other non-salmonid ghr, so-called type I ghr [6]. In contrast, the two isoforms of salmonid ghr previously characterized [[47-49], GenBank:AF403539 and GenBank:AF403540] form a distinct clade amongst other recently cloned non-salmonid type II ghr. Both black sea bream type I and type II ghr have been characterized in ligand binding studies as ghr, not slr, with the use of heterologous (salmon) Prl and Sl [50]. Native hormones have only been used to obtain binding characteristics for the Masu salmon ghr and slr. The Masu salmon slr was found to bind Sl with greater affinity than Gh [5]. The Masu salmon type II ghr bound Gh exclusively [47]. This has led some authors to postulate that type I ghr can phylogenetically be regarded as slr, while type II ghr would be "true" ghr [20]. However, a recent synteny analysis [33] has established that slr is a teleost-specific paralogue of ghr, i.e. it probably appeared by duplication of ghr during the teleost genome duplication event [28], in contrast to Sl, which existed before, but was lost in tetrapods.

The Salmonidae family underwent an additional whole-genome duplication event (4R) about 25–100 million years ago [51]. This gave rise to two closely related salmonid ghr type II isoforms. Only one type of salmonid slr has been found so far, but there could be another closely related isoform which could perhaps differentially bind Slα and Slβ. The long evolutionary history of fish with episodes of genome doubling could have resulted in lineage-specific diversification of ghr and slr, binding Gh and Sl with varying degrees of affinity, and triggering different cellular responses in different groups of fish.

Salmon slr appears to have reduced affinity for Gh, perhaps due to the lack of conserved site B involved in Gh binding, as found for sheep and rat [46]. Salmon slr was found to be less specific in differentiating between Sl and Gh than salmon type II ghr, and an eight-fold concentration of Gh displaced 50% of the Sl bound to slr [5]. This implies that in situations such as fasting, where Gh levels in plasma may become 10-fold higher than Sl, Gh could induce responses via the slr. Further *in vitro *and *in vivo *studies are needed to distinctly characterize both types of receptors in their binding, signaling and biological actions.

### Putative role of Sl and slr in female Atlantic salmon sexual maturation

In salmonids, most studies link Sl to sexual maturation, as plasma Sl levels increase during sexual maturation [7-11] and somatolactotrophs appear activated during this period [12,13]. There are, however, conflicting data as shown in one study on chum salmon spawning migration [14] that found no correlation between plasma Sl and final gonadal maturation. Moreover, Sl function may not be crucial for reproductive success as the Sl-null mutant medaka *ci *can reproduce [20].

A recent study on previtellogenic oocyte maturation in coho salmon [15] found little evidence for a role of Sl during the cortical alveoli or lipid-droplet stage in this species. However, they did find indications that Sl may have a role at even earlier stages of oocyte development, as ovarian *slr *transcripts became downregulated as maturation advanced during the autumn and spring preceding ovulation. Furthermore, tissue screens have shown a relatively high expression level of *slr *transcripts in the gonads of 1 year old coho salmon parr, lesser only to that of liver, visceral fat and muscle [5] and in immature female Atlantic salmon post-smolts (Fig. 9).

In the present study, no change in ovarian *slr *transcripts was found between oocyte oil-drop stage, vitellogenesis, spawning and post ovulation (Fig 6.). This lack of receptor up- and down-regulation contrasts with the pronounced up and down regulation of *Slα *and *Slβ *pituitary transcripts observed that supports the notion that Sl could play important roles during final maturation of female Atlantic salmon. Two important instances of significant increases of both *Slα *and *Slβ *transcripts in the pituitary are found. The most noticeable increase takes place during late vitellogenesis and spawning (September, October and November), with an ensuing down-regulation to baseline levels during ovarian post ovulation in December. The fact that *Slα *and *Slβ *transcripts reach their highest levels at the time of spawning (November) and then drop drastically to baseline levels post ovulation suggests a direct role for *Slα *and *Slβ *in the final stages of ovarian growth and spawning.

Another possible explanation for the observed changes in pituitary *Slα *and *Slβ *during September, October and November (Fig. 6), could be an indirect role for Sl related to maturational changes in plasma calcium and phosphate metabolism during vitellogenesis and spawning. It has been suggested that Sl may play an important role in the increase in plasma calcium that takes place during vitellogenesis [52,53]. At this time, estradiol-17β (E_2_) stimulates hepatic vitellogenin production and mobilizes calcium and phosphate from the environment and/or internal stores to the salmonid oocytes [54].

Somatolactotrophs are activated by low environmental calcium [43] and a study in Baltic Atlantic salmon found that most of the calcium mobilization and incorporation into oocytes occurs in a hypocalcemic environment [54]. Studies have demonstrated that in salmonids not only does vitellogenin correlate with total plasma calcium during the months preceding ovulation [55], E_2 _also correlates with Sl [7,8]. The present study shows that *Slα *correlates positively with E_2 _during post ovulation while *Slβ *correlates positively with E_2 _in August when the GSI increases from 2.8% to 7.9%, and also in November during spawning and post ovulation. During the final months of oocyte maturation, *Slα *and *Slβ *correlate with both gonad weight and GSI. The largest increase in *Slα *and *Slβ *coincides with the sharpest increase in GSI starting in August, which is also the time when calcium-containing vitellogenin is rapidly incorporated into the oocytes. It is also tempting to speculate that the transient peak in Sl observed in February could signal the start of the Ca mobilization that occurs around this time (i.e. onset of exogenous vitellogenesis). The putative role of Sl in calcium mobilization could be in part mediated by PTHrP which incidentally is produced in the vicinity of somatolactotrophs [30,31], mediates the hypercalcemic effect of E_2 _[56] and potentiates E_2_-stimulated vitellogenesis [57]. Sl could also have a direct role in calcium absorption from the water as *slr *expression is high in the gills (but not in the kidney) of Atlantic salmon (Fig. 9) and masu salmon [5]. Likewise, Sl could play a similar role in the mobilization of phosphorous for the incorporation into vitellogenin: Sl increases inorganic phosphate reabsorption by the proximal tubule cells of the kidney in flounder [42].

On the other hand, it has long been suggested [7] that Sl may have a facilitating metabolic role during maturation. The large increase in *Slα *and *Slβ *(September, October and November) coincides with the shift from somatic to gonadal growth that has also previously observed in maturing coho and Atlantic salmon [7,8]. Sl has been found to have lipolytic action in European sea bass [29] and like GH, seems to act as mediator to fasting that maintains lipolytic tonus in temperate fish [40,58-60]. Taken together this could imply a role for Sl in lipid mobilization during sexual maturation in salmonids.

In May, another important transient increase in pituitary *Slα *and *Slβ *transcripts takes place (Fig. 6), prior to transfer from seawater to fresh water. After the transfer, the fish stop feeding as is normal for maturing Atlantic salmon [61]. This transient increase in May is concurrent with the end of primary and secondary yolk formation, when all the fish have reached the tertiary yolk stage. The transient increase in May coincides again both with the first doubling of the GSI and the start of the decline in somatic weight so it is not clear whether the increase in pituitary *Sl *expression is a direct regulatory response linked to ovarian development, or a preparatory response to a stressful life-stage transition which includes both changes in salinity (seawater to freshwater) and nutritional status (voluntary anorexia) during the anadromous spawning migration. However, there is no evidence in salmonids that Sl has a direct freshwater-adapting role [7,8,11,20] and the peak observed during smoltification in coho salmon [8] is believed to be related to its role in energy balance, especially its lipolytic function.

The current study is based on correlations between expression of pituitary *Slα *and *Slβ *and ovarian *slr *transcripts and stage of gonadal development/time during a reproductive cycle. Hence it was not designed to precisely distinguish between different possible roles associated with reproductive development in salmon such as body colour transformations, feed deprivation and anorexia, gonadal growth, spawning and associated metabolic changes. More studies are therefore needed to further clarify the specific roles of Sl during sexual maturation, to elucidate specific functions related to gonadal growth and development, lipolysis, calcium and phosphate metabolisms and body coloration.

## Conclusion

In summary, the aim of the study has been to sequence and phylogenetically characterize Atlantic salmon Slα, Slβ and slr, and to elucidate their potential role in sexual maturation of female Atlantic salmon. The study confirms an increased mRNA expression of *Slα *during gonadal growth and down-regulation during post ovulation, and shows for the first time that *Slβ *expression also changes before, during and after spawning. The increases of both *Slα *and *Slβ *during late vitellogenesis and spawning, and downregulation to baseline levels after ovulation strongly suggests an important role for Sl either directly or indirectly related to reproductive functions in salmon. Among these functions, Sl could be involved in plasma calcium and/or phosphate regulation, lipid metabolism or the regulation of maturationally induced fasting as well as have a direct effect on gonadal development and spawning.

## Competing interests

The authors declare that they have no competing interests.

## Authors' contributions

SB carried out the cloning, phylogenetic analysis, gene expression studies and drafted the manuscript. EA carried out the design of the study, sampling and gene expression studies. GLT carried out the design of the study, fund-raising and sampling. BThB carried out the design of the study and fund-raising. All authors helped to draft the manuscript and read and approved the final version.
